# Co-design and evaluation of an audio podcast about sustainable development goals for undergraduate nursing and midwifery students

**DOI:** 10.1186/s12909-024-06268-3

**Published:** 2024-11-05

**Authors:** Tara Anderson, Patrick Stark, Stephanie Craig, Johanna McMullan, Gail Anderson, Clare Hughes, Kevin Gormley, Jane Killough, Nuala McLaughlin-Borlace, Laura Steele, Sara Lynch, Jesús Sánchez-Martín, Francisco Zamora-Polo, Adam Rodman, Rongrong Li, Gary Mitchell

**Affiliations:** 1 Queen’s University Belfast, School of Nursing and Midwifery, Belfast, UK; 2 Queen’s University Belfast, Queen’s Business School, Belfast, UK; 3 Queen’s University Belfast, Belfast, UK; 4 Departamento de Didáctica de las Ciencias Experimentales y Matemáticas, Facultad de Educación, Universidad de Extremadura, Badajoz, Spain; 5 Departamento de Ingeniería del Diseño, Escuela Politécnica Superior, Universidad de Sevilla, Sevilla, Spain; 6 Harvard University, Harvard Medical School, Boston, USA; 7 School of Nursing, Soochow University, Suzhou, China

**Keywords:** Sustainable development goals, Nursing, Midwifery, Education, Undergraduate nursing education, Undergraduate midwifery education, Podcast

## Abstract

**Background:**

Sustainable Development Goals (SDGs) are universally recognised targets designed to combat poverty, inequality, and climate change. However, there exists limited awareness and understanding of these goals among nursing and midwifery students. To address this knowledge gap, a co-designed audio podcast was introduced as an educational tool to enhance students’ comprehension of SDGs and their relevance to the healthcare profession.

**Methods:**

A prospective study was conducted at Queen’s University Belfast, involving 566 first-year nursing and midwifery students. A 60-minute SDG podcast, co-designed with students and stakeholders, was made accessible within the university’s learning management system. Pre- and post-test questionnaires were administered to assess changes in students’ knowledge levels and attitudes toward SDGs. Additionally, 37 participants engaged in focus group interviews six months after listening to the podcast to explore their experiences and reflections. Quantitative data was analysed using paired t-tests and descriptive statistics, while qualitative data was analysed thematically.

**Results:**

The podcast significantly increased students’ awareness of SDGs and their understanding of the goals’ relevance to their profession and personal lives. Post-test scores showed substantial improvements across all three sub-scales: knowledge, professional relevance, and personal relevance. Moreover, participants rated the podcast as a valuable learning resource with high acceptability, although some expressed uncertainty about replay intentions. Focus group interviews revealed three themes, including 1) More than you know’, which described how participants developed new knowledge and understanding about SDGs, 2) ‘Nurse-Midwife Nudges’, which illuminated how participants made minor changes to their behaviour and 3) ‘Fitting Format’, which highlighted how participants favoured the use of an audio podcast to learn about SDGs.

**Discussion:**

This study demonstrates the potential of audio podcasts as an effective and engaging tool for increasing awareness and understanding of SDGs among nursing and midwifery students. The results suggest that such interventions can positively impact students’ knowledge, attitudes, and behavioural intentions regarding the SDGs. The findings also emphasise the importance of co-design in developing educational resources tailored to the specific needs and preferences of students.

**Supplementary Information:**

The online version contains supplementary material available at 10.1186/s12909-024-06268-3.

## Introduction

Sustainable Development Goals (SDGs) are universally agreed targets which aim to reduce inequalities, end poverty, and tackle climate change [1]. The 17 SDGs which cover a range of issues including education, gender equality, and clean water, have been adopted by all United Nations (UN) member states as part of the 2030 Agenda for Sustainable Development [1]. According to the 2023 Progress Report, progress on achieving these goals has been limited due to factors including the COVID-19 pandemic and Russia’s invasion of Ukraine [2]. This has disproportionally impacted developing countries [2] and has highlighted the urgency of addressing these issues.

Nurses and midwives, which make up almost half of the global health workforce, have a crucial role to play in achieving the SDGs [3]. Helping people to achieve optimal health, nurses and midwives frequently address social determinants of health (SDH), a critical component of the SDGs. Health inequalities are most often the result of SDH [4], for example poverty, social status, education, employment, and food security. The understanding of the links between these factors and individual and population health within the nursing and midwifery profession demonstrates the importance of the profession to achieving the SDGs [4].

Globally, the nursing and midwifery profession has been working to address the SDGs in many ways. For example, creating and delivering literacy workshops and education programs on self-assessment for health screening in the Appalachian communities in the United States (US) [5]. Other US examples include leading an environment-community alliance and establishing green initiative committees within hospitals [5]. A recent publication of the Royal College of Nursing (RCN) highlighted some of the many examples of the contribution of nurses and midwives to achieving the SDGs within the UK [6]. Some of which included tackling homelessness in hospital and community settings, outreach work with vulnerable young people at risk of being exploited and those involved in gangs, and addressing health inequalities among mothers and babies from ethnic minority groups [6].

Despite the importance of nursing and midwifery to the SDGs, literature on the topic is limited. A recent review of publications relating to nursing and midwifery and the SDGs found most to be editorial, discussion pieces [7]. However, the review did highlight a common theme within the literature which recommended the integration of SDG content within undergraduate nursing and midwifery training [7]. Many nurses and midwives are not aware about what the SDGs are and how they can contribute [8], and limited studies exist on the educational interventions that support nursing student understanding about SDGs [9]. Recent research has also indicated that this is common and that most university students lack knowledge and awareness about the SDGs [10]. The present study aims to address research gap through co-designing, implementing, and evaluating an audio podcast, based on the SDGs, to nursing and midwifery students.

Podcasts are becoming a widely used learning resource within nursing education with students reporting their utility as a learning tool, revision aid, and in helping to understand course materials [11–14]. Podcasts represent an engaging way to meet the demands of nursing education and may help to increase student engagement and facilitate revision of material [15, 16]. In addition, the use of podcasts as a learning tool in higher education has been suggested to complement a constructivist approach to learning, in which listeners may internalise their knowledge and reflections and apply this within their own experiences [17]. This approach is especially relevant, with increasing nursing and midwifery student intentions to act on the SDGs a primary aim of this study.

The present study, therefore, uses a co-designed audio podcast which provides explicit education about the UN’s SDGs and how each of these 17 goals applies to nursing and midwifery students including practical suggestions for how students can help to achieve the SDGs. This podcast was co-designed with nursing and midwifery students. This involvement of students in the design of the educational intervention has been shown to help provide an insight into the functionality of an intervention and how this can best support learning [18, 19].

## Method

### Study aim

To assess the effectiveness of a co-designed audio podcast about SDGs as an educational tool.

### Objectives

1. To quantify the change in knowledge levels among nursing and midwifery students regarding SDGs before and after listening to the co-designed audio podcast, as measured by pre- and post-test questionnaires.
2. To explore the impact of the audio podcast on students’ perceptions and attitudes. Specifically, assessing the impact on students’ reflection on the SDGs and how this learning was applied to their own personal and professional experiences.
3. To gather students’ feedback on the podcast as a learning resource.

### Design/ setting/ population

A prospective descriptive exploratory approach was used with both a quantitative and qualitative element. A quantitative pre-test/post-test design was used to compare student knowledge and attitudes to the UN’s Sustainable Development Goals before and after listening to the podcast. All students enrolled in their first year of nursing (including adult, children, mental health, and learning disability fields of nursing) or midwifery in Queen’s University Belfast were eligible for inclusion in this study. The study was conducted using convenience sampling of this population (*n* = 670).

Participants were also invited to participate in a 30-minute online focus-group interview (via MS Teams) with a member of the research team to discuss how they translated learning from the podcast. All participants who had completed the quantitative element of the study were eligible for inclusion in the follow-up focus groups. Those who expressed interest in taking part were followed up with by a member of the research team. These participants then took part in one of four focus group interviews six months after listening to the original podcast.

### Intervention

The 60-minute podcast was recorded by service users, carers, nursing students, and midwifery students. A copy of the podcast is available upon reasonable request from the corresponding author. The podcast covers each of the 17 SDGs, how each applies to nursing and midwifery students, and practical suggestions of what students can do to help achieve them. For example, for SDG1 (poverty), students were provided with a case-study about how nurse family partnerships in urban neighbourhoods can help support families with low income. For SDG5 (gender), the podcast considered how nurses and midwives could reduce HIV stigma. For SDG7 (energy), the impact of air pollution and rates of pneumonia was discussed and for SDG12 (Consumption/Production), the implications of inappropriate disposal of syringes were considered.

The audio podcast was co-designed by a special interest group formed by students and staff at the university. Members of the co-design group scripted the audio podcast to ensure that each SDG would be discussed for approximately 3 min by a current student nurse, student midwife, service user or carer.

### Data collection

All year one nursing and midwifery students were given access to the podcast which was uploaded as an MP3 audio file within the Canvas Learning Management System used by students for their university course. Students could listen to the podcast at any convenient time within a 30-day period and complete the voluntary pre- and post- test questionnaires before and after listening. Students had the flexibility to listen to the podcast on their phone, with headphones, on a computer, or any preferred device. Additionally, they could choose to complete the podcast in one sitting or break it into smaller segments, depending on their schedule and preference. All students could choose to listen to the audio podcast without participating in the research study. Further, all students were instructed that failure to participate in the study would not affect their grades.

The pre- and post-questionnaires were also embedded within a Canvas module page. A link to this page including the participation information sheet was emailed to all prospective participants. The pre-questionnaire recorded demographic details (gender, age, field of study). This was followed by a 42-item SDG questionnaire (Supplementary File 1) based on a previously published questionnaire [10]. The first eight items of this questionnaire provided general statements about SDGs (e.g., I know what the Sustainable Development Goals are) while the subsequent 34 items provided statements related to the relationship between the SDGs and the nursing/midwifery profession from students’ point of view and lifestyle (e.g., I consider the profession for which I am training to be related to: Poverty reduction). The SDG questionnaire recorded responses on a 5-point Likert scale ranging from 1, strongly disagree, to 5, strongly agree.

The post-questionnaire repeated this questionnaire and included an evaluation of the podcast (Supplementary File 2). This consisted of a 6-item questionnaire which measured the acceptability of the SDG podcast (e.g., The SDG podcast is a good learning resource). Responses were also measured on a 5-point Likert scale ranging from 1, strongly disagree, to 5, strongly agree.

Participants were also invited to attend a focus-group session following listening to the podcast. This was conducted by two members of the research team online via Microsoft Teams, based on a pre-determined interview guide that was designed by the authors (Supplementary File 3).

### Ethics

Queen’s University Belfast, Faculty of Medicine, Health and Life Sciences Research Ethics Committee granted ethical approval for this study (Ref: MHLS22_113) after considering benefits and risks and ensuring participants autonomy would be respected. All methods were performed in accordance with the Declaration of Helsinki. The study began in September 2022 and concluded in August 2023.

In this study, power imbalances between participants and researchers were addressed through several strategies. Informed consent was prioritised by providing participants with detailed information about the study’s purpose and their rights, ensuring they could make informed decisions about participation. Participation was voluntary, with clear assurances that withdrawal at any time would not affect students’ academic standing or relationships with faculty. Anonymity and confidentiality were strictly maintained to protect participants’ identities. Additionally, the co-design of the podcast allowed students to contribute to the content, further reducing power differentials by fostering a sense of ownership in the process.

### Consent/ recruitment

All prospective participants were emailed the participant information sheet and a link at which they could complete the study. A participant information sheet was first presented outlining the details of the study. Participants gave their consent to complete the questionnaire when they actively accessed the survey web links and ticked a consent statement. Participants provided their email address for the purposes of linking the pre- and post-questionnaires. Students who attended a follow-up focus group session provided informed consent after viewing a new specific participant information sheet. All participants were permitted to withdraw from the study at any stage, without giving any reason and information about these processes was provided within both participant information sheets.

### Data analysis

All data was uploaded to SPSS version 29 for statistical analysis. The research team were the only individuals with access to the data. All data collected during this study complied with the General Data Protection Regulations (GDPR).

Quantitative data was analysed using scores calculated as sum of Likert scale responses for each of the three SDG sub-scales. The minimum and maximum scores for the 4-item Awareness subscale were 5 and 20 respectively. The minimum and maximum scores for the 17-item Professional and Personal subscales were 17 and 85 respectively. Descriptive statistics were calculated for pre-test and post-test scores. A series of paired t-tests were conducted to examine the change from pre-test to post-test for each of the three pairs of subscales.

In total, three significance tests were conducted during this study. A Bonferroni correction was applied to the alpha value when determining the statistical significance of the results of these analyses to reduce the risk of false positives associated with multiple comparisons. Alpha (0.05) was divided by the total number of comparisons in this study [3] to give an alpha value of *α* = 0.017. Results of the pairwise comparisons in this study, therefore, were only considered to be statistically significant if their associated *p*-value was 0.017 or below.

Responses to the technology acceptance questions asked at the post-test time point were analysed descriptively as percentages of response categories for each of the five Likert responses (strongly disagree to strongly agree).

Qualitative data was audio recorded, transcribed verbatim and analysed using thematic analysis. The analysis process employed the six-step thematic analysis framework by Braun and Clarke (2006) [20]. This method of analysis facilitated familiarity with the data and facilitated the research team to recognise themes as they emerged.

## Results

### Quantitative

In total, 566 participants (Table 1) were recruited to evaluate the impact of a sustainable development goals podcast on year one nursing and midwifery students’ knowledge and attitudes about the goals. This represented a response rate of 84.48% of the total sample (*n* = 670). Most participants were female (93.46%) and studying adult nursing (63.25%). Age ranged from 18 to over 50 with the 18–21 age group representing the majority (67.14%).

**Table 1:** Participant descriptive statistics

		*N*	%
Gender	Female	529	93.46%
	Male	23	4.06%
	Non-Binary	1	0.18%
	Prefer Not to Say	1	0.18%
	Missing	12	2.12%
Age	18–21	380	67.14%
	22–25	58	10.25%
	26–30	31	5.48%
	31–34	38	6.71%
	35–40	27	4.77%
	41–45	10	1.77%
	46–50	6	1.06%
	50+	4	0.71
	Missing	12	2.12%
Field	Adult Nursing	358	63.25%
	Children & Young People’s Nursing	85	15.02%
	Learning Disability Nursing	31	5.48%
	Mental Health Nursing	52	9.19%
	Midwifery	28	4.95%
	Missing	12	2.12%

### Missing data

Primary analysis was possible for *N* = 525 cases out of a total of *N* = 566 due to missing data. If a participant was missing either pre-test or post-test, they were not able to be included in the primary analysis paired comparisons, but their non-missing data was included in the descriptive statistics. Missing data occurred due to a participant not sitting either the pre-test or post-test or due to participants not supplying a correct identifier (email address) to allow their pre-test and post-test data to be matched. Table 2 shows the level of missing data at pre-test, post-test and analysis.

**Table 2:** Missing data

	Missing *n* (%)
Pre-test data	12 (2.12%)
Post-test data	29 (5.12%)
Paired comparisons	41 (7.24%)

### Preliminary analysis

Descriptive statistics (Table 3) show that post-test scores were higher than pre-test scores for all three of the subscales. The difference between these two scores were statistically significant as indicated by three paired samples t-tests shown in Table 4 (*p* < 0.001 and below the Bonferroni-corrected alpha cut-off of *p* = 0.017).

**Table 3:** Pre-test (*n* = 554) and post-test (*n* = 537) mean scores for sub-scales

	Pre-test Mean	Pre-test Std. Deviation	Post-test Mean	Post-test Std. Deviation
Awareness	9.78	3.58	17.21	2.20
Professional	58.79	10.13	75.06	8.78
Personal	56.07	10.75	69.38	10.38

**Table 4:** Paired comparison t-tests for pre-test and post-test sub-scale scores (*N* = 525)

		Mean increase from pre-test to post-test	Std. Deviation	t^a^	Df^b^	Sig. (2-tailed)
Pair 1	Post-test awareness and pre-test awareness	7.44	3.79	45.01	524	< 0.001
Pair 2	Post-test professional and pre-test professional	16.50	10.68	35.39	524	< 0.001
Pair 3	Post-test personal and pre-test personal	13.42	10.40	29.57	524	< 0.001

^a^ paired samples t-test

^b^ degrees of freedom

Overall, participants showed significantly higher awareness of SDGs and their professional and personal responsibilities relating to these after listening to the SDG podcast.

### Technology acceptance

Responses to the items about technology acceptance for the SDG podcast are shown below in Table 5. Overall, there was a clear majority positive response (either agree or strongly agree) to all items except for “I will listen to the SDG podcast more than once” which showed 30.5% neutral responses alongside its otherwise positive majority (58.1% either agree or strongly agree).

**Table 5:** Acceptability of the SDG podcast

	Strongly disagree (%)	Disagree (%)	Neutral (%)	Agree (%)	Strongly agree (%)
The SDG podcast is a good learning resource	0.6%	0.4%	5.2%	48.0%	45.8%
The SDG podcast was straight-forward & easy to understand	0.2%	0.7%	6.7%	42.8%	49.5%
The SDG podcast met my learning needs	0.4%	0.9%	9.3%	50.8%	38.5%
I would recommend the SDG podcast to others	0.2%	0.2%	10.2%	49.3%	40.0%
The duration of the SDG podcast was appropriate	0.2%	4.8%	13.6%	48.2%	33.1%
I will listen to the SDG podcast more than once	1.7%	9.7%	30.5%	41.9%	16.2%

### Qualitative results

Forty-eight participants indicated that they would be interested in taking part in a focus group interview as part of the post-test. Of these, 37 participants consented and took part in one of four focus group interviews at the period of six months after listening to the original podcast. The remaining 11 participants did not respond to two follow-up emails from the team regarding their participation in focus groups and were therefore it was assumed they were no longer interested in participating.

Following thematic analysis, three themes were identified by the research team, and these were (1) ‘More than you know’, which described how participants developed new knowledge and understanding about SDGs, (2) ‘Nurse-Midwife Nudges’, which illuminated how participants made minor changes to their behaviour and (3) ‘Fitting Format’, which highlighted how participants favoured the use of an audio podcast to learn about SDGs. A copy of the consolidated criteria for reporting qualitative studies (COREQ) is available in Supplementary file 4.

### More than you know

Entering their first year of the program, student participants found themselves immersed in learning the fundamentals of care, clinical skills, and understanding the importance of professionalism associated with their role. Many participants therefore expressed initial feelings of scepticism, surprise, and disappointment about the prospect of studying sustainable development goals within their programme as noted in the excerpt that follows. *“I was confused to be honest*,* at the time I remember thinking – why do I need to know this now? We [nursing and midwifery students] were about to embark on placement and I thought this was something for later [in the programme]*,* you know after we got the basics” [FG2*,* P5].*

After listening to the podcast, participants unanimously agreed that the content was important learning and surpassed any initial uncertainty about its suitability. Their audio learning began as they explored various SDGs, each focusing on a multifaceted aspect of global sustainability. One participant stated, *“I never thought nursing was connected to all this. It felt like a broadening of perspective.” [FG1*,* P2].* The learning experiences participants discussed were broad as they discovered the intricate interplay between health and the broader global landscape. For instance, participants discussed how they learned about SDG 12, Responsible Consumption and Production, understanding the environmental impact of healthcare practices. Another cited example came with SDG 7, Affordable and Clean Energy, as students explored how healthcare facilities could embrace sustainability. A student reflected, *“I never considered how the way we handle waste in hospitals impacts the environment. It was not on my radar. Not at all.” [FG1*,* P3].*

This newly acquired knowledge was discussed extensively during focus group interviews with participants. Overall, participants reflected that they began to appreciate the importance of the information they had learned about as noted here,

*“Now I know*,* I can’t unknow. And that knowledge is a tool for change I guess*,* which is why we get it at the beginning [of the programme] I suppose.” [FG3*,* P11].* Additionally, another participant stated, *“I would go as far to say that sustainability or looking after the planet or whatever…and everything else in the sustainable development goals*,* that are not explicitly health-related*,* was more than you know as a [nursing or midwifery] student.” [FG4*,* P1].*

In addition to their experiences of learning about SDGs through the podcast, participants also discussed how the acquisition of this new knowledge could become a catalyst for change or empowerment. Participants across all four focus groups discussed how the education could inspire students to be advocates for sustainable practices within their profession and within their local communities as stated here. *“Gosh*,* yes*,* I can imagine there would be some of us [student nurses or midwives] that could be drivers for change. You know*,* Queen’s [the University] has clubs and societies for green initiatives and sustainability and all*,* so there is a good place to start if we wanted to start something [referring to an initiative]”. [FG3*,* P8].* Another participant shared, *“It’s not just about what I do as a nurse; it’s about what we do collectively to make a difference in the world and that was very inspiring.” [FG1*,* P2].*

In summary, the theme *“More Than You Know”* discusses the learning journey of nursing and midwifery students as most navigated from scepticism to enlightenment through the SDG podcast. Participants demonstrated that there was a realisation that there was connection between their profession and global sustainability. Participants perceived that this knowledge was important and could motivate or empower their peers in the future.

### Nurse-midwife nudges

Following on from theme one, participants discussed how the audio podcast led to subtle yet impactful shifts in their behaviour which regards to the sustainable development goals. These minor changes, driven by increased knowledge, underscored the potential for nurses and midwives to become agents of positive transformation in both their professional roles and personal lives.

Many participants that listened to the podcast, noted how they embraced a spectrum of small adjustments in their daily practices. These related to a range of sustainable development goals, including SDG 7, *“I started turning off lights and equipment not in use during my shifts*,* realising how much energy we could save at the nursing home”* [FG1, P7], SDG12, *“I would be quite good at doing my bit for the environment anyway*,* but one thing I did do was to create a small recycling corner in our break room at the church*,* making it easier for GB [Girl’s Brigade] to recycle paper and plastic. It was a small change really”* [FG4, P9], SDG 5, *“I signed up to become one of the student representatives at the university EDI [equality*,* diversity and inclusion] team [Anthea SWAN subgroup at School of Nursing and Midwifery]”* [FG2, P4] and SDG 4, *“Our tutorial group designed a health promotion poster to create awareness about health disparities in people living with learning disabilities”* [FG1, P6]. These changes in behaviour illustrated the potential for sustainable development education to influence the mindset of future healthcare professionals.

Beyond individual changes, nursing and midwifery participants also recognised their capacity to influence others. Participants collectively spoke about ways in which they actively shared what they learned with others in a view to influence their behaviour in relation to SDGs. This resulted in the following, for SDG 6, *“My boyfriend would have spent like 30–40 minutes in the shower and now*,* I am like*,* you’ve got 3–4 minutes. He is just as clean to be fair [laughs]*,* but he is saving literally ten times the water*,* right?”* [FG3, P2], SDG 7, *“I have small people [children] at home*,* so I am always giving out to them about turning off lights*,* turning off phone chargers or TVs – you name it”* [FG 4, P4] and SDG 3, *“I am the one in the group who is always making us go on walks or swims or whatever…I just think we all need to be reminded to look after our physical and mental health”* [FG1, P8]. Few participants spoke about how they encouraged their peers in healthcare settings to make positive changes with the SDGs in mind, however it was encouraging that many participants explicitly noted how they were supporting attainment of SDGs.

While there was good evidence of behaviour change noted by participants, there were others within focus groups that had fewer positive experiences regarding influencing behaviour change. In three of the four focus groups, participants noted how they had encountered challenges. The most common reasons for this included things like (1) unwillingness to change daily habits and resistance to adopting new, more sustainable practices, or (2) preference for fast food and packaged snacks for convenience because less effort involved in preparation and cleanup, or (3) scepticism about the urgency and reality of climate change due to perception it as a distant or exaggerated issue and (4) too busy with work or personal commitments to dedicate time to recycling, believing it is easier to dispose of items in a single bin. This was reflective in the following statements, *“I know some of the students [peers] are using foodbanks and can’t really afford to buy fresh*,* locally sourced foods”* [FG1, P5], *“I think we all have a role to play*,* but to be honest*,* my mum and dad are set in their ways and don’t know anything about sustainability – that is definitely a barrier”* [FG3, P2] and *“To be honest*,* all we can really do is nudge people in the right direction. For me*,* that is much easier to do now that I know about this kind of stuff”* [FG4, P3].

In summary, the theme “*Nurse-Midwife Nudges”* illuminates how podcast listeners were capable of subtly influencing, or nudging, themselves and others to implement minor adjustments that would contribute to the benefit of Sustainable Development Goals. Participants also noted that despite this encouragement, they were at times met with challenges from others about implementing change.

### Fitting format

The previous two themes explored participant experiences of the audio podcast in the context of knowledge, self-efficacy and behaviour change. Further to this, an important third theme emerged from the focus groups, and this was about the significance of audio podcasting as an instrumental method for delivering accessible education about the SDGs.

Participants uniformly acknowledged podcasting as an effective medium for SDG education. Their consensus emphasised the value of incorporating diverse voices, including those of 17 individuals affected by the goals, including current nursing students, midwifery students and members of their school’s Patient and Carer Education Partnership: (https://www.qub.ac.uk/sites/PatientandCarerEducationPartnership/). One participant expressed their appreciation of the podcast, noting, *“It was good that there were several speakers on the podcast. It made the content more relatable*,* and I found it intriguing to see how different SDGs can impact people globally. It has made me more aware of the goals”* (FG3, P2).

Despite the recognition of the podcast’s comprehensive approach, concerns were raised about its 60-minute duration. Participants suggested that a shorter format could enhance engagement, with one stating, *“I tend to lose a bit of interest after about 15 or 20 minutes. If it was around 40 minutes and in two parts*,* I could engage with it more effectively*,* but maybe that is just me”* (FG2P1). Another participant shared a similar comment, suggesting that *“The podcast was really good; however*,* I believe it could have been shortened to around 30 minutes or so”* (FG1, P7).

In addition to duration, participants highlighted variations in how they engaged with the podcast, emphasising the need for guidance in future. One participant noted, *“I think our class struggled a bit in knowing how we were meant to use the podcast. Some listened to it while doing other things*,* but some of us treated it like a lecture and took notes. Maybe*,* a bit of advice on how to get the best out of it would be my recommendation”* (FG2, P2). Others expressed the desire for supplementary resources, with a participant stating, *“I listened to the podcast twice*,* I actually did geography at A-Level [UK Pre-University Qualification undertaken at secondary school]*,* so already knew a lot of this*,* but not from the health professional angle. I don’t know*,* I suppose after listening I just wanted more information on specific SDGs or like something to read or like a report or summary sheet. I guess*,* recommendations for supplementary reading or videos would be helpful*,* you know for those who want it”* (FG3, P6).

The production quality of the SDG awareness podcast emerged as a pivotal aspect contributing to its effectiveness. Participants praised elements such as narration, background music, and different segments. They perceived these features as instrumental in capturing their attention and fostering a professional atmosphere. One participant remarked, *“Hearing a different speaker for every SDG was cool*,* having different segments too about like*,* what SDGs were or like what the university is doing to meet SDGs kept me interested. It felt like a professional podcast”* (FG1, P8). Another participant appreciated the inclusion of nursing and midwifery students in the production, stating, *“Having actual nursing and midwifery students in the interviews was brilliant. It resonated with me*,* as they are like me*,* and speak in a way that I can understand”* (FG4, P6).

In summary, participants perceived the SDG awareness podcast as a valuable educational tool, offering diverse perspectives on each goal. While praising its comprehensive nature, they suggested adjustments to enhance engagement, emphasising the importance of clear guidance and supplementary resources. The high production quality emerged as a key factor in the podcast’s success, contributing to a professional and engaging learning experience.

## Discussion

Audio podcasts have emerged as a valuable tool, providing educational and entertaining content that is easily accessible [21]. This medium enables experts to disseminate knowledge to a broad audience, and its pre-recorded format ensures flexibility, allowing listeners to engage with the material at their convenience [22]. The versatility of podcasts accommodates diverse study habits, enabling students to learn at their preferred pace while multitasking [21, 22]. Recent research even suggests that podcasts may enhance knowledge retention compared to traditional reading methods [21, 22]. Despite the increasing popularity of podcasts as educational resources, particularly in healthcare, there remains a notable lack of comprehensive guidance on the effective development and evaluation of such content [23, 24]. The central objective of this study was to introduce an educational resource aimed at supporting students to develop a deeper understanding of sustainable development goals for future nurses and midwives. The current study adds to the existing body of literature that illustrates how audio podcasting can support healthcare profession students to have greater awareness, self-efficacy and behaviour change following this type of educational intervention [25].

The health education podcasting landscape is notably diverse, encompassing various formats and affiliations. Research conducted by Zhang and colleagues investigated the 100 most popular medical podcasts in the United States, revealing a rich variety of didactic methods, including those aligned with different levels of Bloom’s taxonomy. Notably, a substantial portion of these podcasts’ targets physician education, catering to a spectrum of medical learners [25]. The authors have been unable to find an audio podcast that discusses SDGs with explicit consideration to future nurses or midwives.

While there exist gaps in comprehending the optimal use and effectiveness of podcasts, their potential to reach a broad audience is evident. This audience spans healthcare students, newly qualified professionals, and practicing clinicians. The versatility of podcasts positions them as powerful tools for disseminating innovative practices and bridging knowledge gaps across diverse healthcare settings, including primary, secondary, and tertiary care [26, 27]. As healthcare education undergoes continuous evolution, podcasts emerge as a dynamic medium capable of fostering positive learning environments and sharing essential cultural competencies [26]. Considering their adaptability and ability to cater to the evolving needs of modern clinical education, podcasts stand out as indispensable components of the educational landscape [26] and this study demonstrates that their medium is also likely to be an effective way to provide education about sustainable development goals.

In the current study, the SDG podcast facilitated increases in post-test scores on each of the three distinct sub-scales related to the SDGs compared to their pre-test levels. Sub-scale one measured knowledge of SDGs and sources of information. Scores on this sub-scale indicated nursing and midwifery students showed very little awareness of SDGs prior to listening to the podcast. These baseline results are similar to results obtained with university students in Spain a few years prior [10]. It is promising, however, that awareness scores significantly increased following listening to the podcast, adding to the evidence-base for podcasting as an educational tool for nursing and midwifery students [12–14].

Participants also showed increased scores following listening to the podcast on the professional and personal sub-scales. These measured the relationship between the SDGs and the profession for which they are training (professional), and their lifestyle (personal). Increased scores, therefore reflected increased awareness and beliefs that SDGs are impacted by their profession and lifestyle. Due to the crucial role nurses and midwives can play in achieving the SDGs [3, 4], it is positive that students’ point of view on these factors is favourable.

The primary goal of the use of this podcast for nursing and midwifery education was to facilitate students’ reflection on the SDGs and apply this learning to their own experiences within their profession. Participants reported increased awareness of the role of their profession and lifestyle in relation to the SDGs. This is in line with the pedagogical approach associated with constructivist theory [17] as podcast-based teaching lead to increased reflections on how the SDGs are relevant to student’s own experiences, both professionally and personally. This was further illustrated during focus group interviews, whereby students discussed how their increased awareness of SDGs led to changes in behaviour.

### Recommendations

The present study represents a novel approach to incorporating SDG education within nursing and midwifery education. This may help to address the current lack of awareness of the SDGs and how nurses and midwives can contribute to achieving them [7, 8]. This study has demonstrated that a co-designed educational podcast can help to facilitate increased awareness of the SDGs and how these relate to individuals’ professional and personal lives. Co-design is particularly important, as incorporating the student voice ensures the intervention is relevant and appropriate for their peers [28, 29]. Given the high levels of acceptability reported for the podcast adding to the previous evidence-base for the benefits of podcasting [11–13, 17]. SDG podcasting may prove a viable alternative to face-to-face teaching to enable incorporation of this education within demanding nursing and midwifery teaching. Within this study, the podcast was designed specifically for year one students, and future interventions will also need to be developed for subsequent years, incorporating approaches such as face-to-face teaching, group work, assessments, or other asynchronous interventions like podcasts, e-learning, and serious games.

### Strengths, limitations and future research

This study is novel, and to the best of our knowledge the first to investigate the use of co-designed podcast-based SDG education with undergraduate nursing and midwifery students. The podcast led to significant improvements in student’s awareness of SDGs which is promising as nurses and midwives have an important role to play in addressing these. In addition, a large sample of participants completed this study (*n* = 525), representing a large cohort of nursing and midwifery students. However, this sample was from one university in Northern Ireland which may limit the generalisability of the findings. Future research may benefit from comparing results between larger, more varied cohorts. A limitation of this study is that, due to the nature of the intervention, it is not possible to confirm whether all students listened to the entirety of podcast, despite it being a course requirement for their learning. However, it is likely that they did, as participation in the research was voluntary, reducing the incentive to falsify responses in the post-questionnaire. The present study also did not collect any information on previous experience with the SDGs which may have been a confounding variable. Future research may benefit from incorporating this factor. Additionally, further research comparing the podcast to another teaching method such as a lecture may be beneficial. While it is important to acknowledge that this study demonstrated an increase in awareness of the Sustainable Development Goals (SDGs) among nursing and midwifery students, further research could enhance the validity of the podcast as an educational tool. Specifically, conducting assessments to measure participants’ knowledge of the SDGs after listening to the podcast would provide data about its effectiveness. A follow-up study could involve pre- and post-assessments to quantify knowledge gains, thereby reinforcing the podcast’s role in promoting understanding and engagement with SDGs in nursing education. Finally, long-term follow-up would facilitate knowledge retention to be investigated as well as examples of putting learning into practice.

## Conclusion

Podcasting represents a low-cost, modern method of teaching within undergraduate nursing and midwifery. There is growing evidence-base that podcasts are both feasible, and acceptable to student populations including healthcare students. The present study adds to this evidence-base, and incorporates theory-based, co-design of the educational intervention. Based on the positive results, this podcast has been integrated into pre-registration education for both nursing and midwifery students at the institution of origin, with no further modifications made. Results highlight that the SDG podcast can increase both awareness of the SDGs and how these relate to the student’s profession and personal life. This is promising given the current lack of knowledge of the SDGs despite the importance of nursing and midwifery to addressing the goals.

## Supplementary Information

Supplementary Material 1.

Supplementary Material 2.

Supplementary Material 3.

Supplementary Material 4.

## Data Availability

The datasets used and/or analysed during the current study are available from the corresponding author on reasonable request.
