# Supplementary material for: Co-design and evaluation of an audio podcast about sustainable development goals for undergraduate nursing and midwifery students
Source: BMC Med Educ. 2024 Nov 5;24:1253. doi: 10.1186/s12909-024-06268-3 (PMC11536588; doi:10.1186/s12909-024-06268-3)
Supplement: Supplementary file 1 — Supplementary Material 1. [file 12909_2024_6268_MOESM1_ESM.docx]

Supplementary File 1 – 42-item SDG questionnaire from Zamora-Polo et al., 2019

**Sustainable Development Goals Knowledge Questionnaire (Zamora-Polo et al. 2019)**

**Respondents to indicate the degree of agreement or disagreement with the following statements. (1)Strongly disagree, (5)Strongly agree.**

1. I know what the Sustainable Development Goals are.

2. I know the countries to which the Sustainable Development Goals are addressed.

3. I know the time horizon for which the Sustainable Development Goals are designed.

4. I know the number of Sustainable Development Goals and could indicate one of their goals.

5. I have received information about the Sustainable Development Goals by email and/or Social Networks.

6. I have received information about the Sustainable Development Goals from the traditional media (press, radio and/or television)

7. I have received information about the Sustainable Development Goals in formal education (high school, university. ect.).

8.I have received information about the Sustainable Development Goals in informal training (e.g., workshops of NGDOs, actions of the University Cooperation Office, etc. ).

**I consider the profession for which I am training to be related to:**

9. Poverty reduction

10. Hunger reduction

11. Health care and wellness

12. Quality education

13. Gender equality

14. Access to clean water and Sewerage

15. Accessible and non-polluting energy

16. Decent work and economic growth

17. Industry, innovation and infrastructure

18. Reducing inequalities

19. Creating sustainable cities and communities

20. Responsible consumption and production

21. Weather care

22. Care of underwater life

23. Care for life in terrestrial ecosystems

24. Peacebuilding, justice and corruption-free institutions

25. Building alliances to achieve the above goals

**I think my lifestyle has an impact on the following aspects:**

26. Poverty reduction

27. Hunger reduction

28. Health care and wellness

29. Quality education

30. Gender equality

31. Access to clean water and Sewerage

32. Accessible and non-polluting energy

33. Decent work and economic growth

34. Industry, innovation and infrastructure

35. Reducing inequalities

36. Creating sustainable cities and communities

37. Responsible consumption and production

38. Weather care

39. Care of underwater life

40. Care for life in terrestrial ecosystems

41. Peacebuilding, justice and corruption-free institutions

42. Building alliances to achieve the above goals
