# Supplementary material for: Co-design and evaluation of an audio podcast about sustainable development goals for undergraduate nursing and midwifery students
Source: BMC Med Educ. 2024 Nov 5;24:1253. doi: 10.1186/s12909-024-06268-3 (PMC11536588; doi:10.1186/s12909-024-06268-3)
Supplement: Supplementary file 2 — Supplementary Material 2. [file 12909_2024_6268_MOESM2_ESM.docx]

Supplementary Material 2 – 6-item acceptability questionnaire

**Acceptability of the SDG Podcast Questionnaire**

**Respondents to indicate the degree of agreement or disagreement with the following statements. (1)Strongly disagree, (5)Strongly agree.**

1. The SDG podcast is a good learning resource

2. The SDG podcast was straight-forward & easy to understand

3. The SDG podcast met my learning needs

4. I would recommend the SDG podcast to others

5. The duration of the SDG podcast was appropriate

6. I will listen to the SDG podcast more than once
