# Supplementary material for: Co-design and evaluation of an audio podcast about sustainable development goals for undergraduate nursing and midwifery students
Source: BMC Med Educ. 2024 Nov 5;24:1253. doi: 10.1186/s12909-024-06268-3 (PMC11536588; doi:10.1186/s12909-024-06268-3)
Supplement: Supplementary file 3 — Supplementary Material 3. [file 12909_2024_6268_MOESM3_ESM.docx]

Supplementary Material 3 – Focus group interview guide

**Focus-Group Interview Guide**

The purpose of this focus-group interview is to discuss how the podcast helped inform student nursing practice during placements. There are a few areas I would like to discuss about your experience. If any participant wishes to stop or withdraw from the interview at any time, please let me know and the interview will be stopped.

***Topic Areas:***

**What were your overall impressions of the podcast?**

- What did you enjoy most about the podcast?
- What did you enjoy least about the podcast?
- How did this podcast influence or change your knowledge about SDGS?
- How did this podcast influence or change your social behaviour?
- How did this podcast influence of change your behaviour within clinical practice?

**What are the limitations of the podcast?**

- How might the podcast be improved for your colleagues in the future?
- What were the challenges of applying your learning from the podcast in practice?
- Did the podcast support your role as a nursing student or nursing midwife?

Thank you for your time, this has been very helpful and I would be extremely interested in any other thoughts or feelings you have and would like to share to help me better understand your experience or is there anything you would like me to go back to?
