# Supplementary material for: Co-design and evaluation of an audio podcast about sustainable development goals for undergraduate nursing and midwifery students
Source: BMC Med Educ. 2024 Nov 5;24:1253. doi: 10.1186/s12909-024-06268-3 (PMC11536588; doi:10.1186/s12909-024-06268-3)
Supplement: Supplementary file 4 — Supplementary Material 4. [file 12909_2024_6268_MOESM4_ESM.docx]

**Supplementary File 4**

Consolidated criteria for reporting qualitative studies (COREQ): 32-item checklist.

| **No** | **Item** | **Guide questions/description** |
| --- | --- | --- |
| **Domain 1: Research team and reflexivity** |  |  |
| Personal Characteristics |  |  |
| 1. | Interviewer/facilitator | All interviews will be facilitated by both Dr Gary Mitchell (GM) & Miss Stephanie Craig (SC) |
| 2. | Credentials | GM & SC both have expertise in qualitative research methods and qualitative data collection (including semi-structured interviewing and focus-group interviewing). |
| 3. | Occupation | GM is a Reader at Queen’s University Belfast in Northern Ireland. SC is a Teaching Assistant at Queen’s University Belfast & in her final year of PhD study. |
| 4. | Gender | GM is male. SC is female. |
| 5. | Experience and training | GM & SC are registered nurses with considerable experience in higher education. |
| Relationship with participants |  |  |
| 6. | Relationship established | GM & SC teach on the Undergraduate Nursing Programme where students were recruited. |
| 7. | Participant knowledge of the interviewer | Participants would have been aware of who was conducting the focus groups as these were detailed in the information sheet and consent forms. |
| 8. | Interviewer characteristics | GM & SC are registered nurses with expertise in the topic area. GM & SC also have a strong awareness in the context (e.g., design, implementation and evaluation of podcasts in higher education). |
| **Domain 2: study design** |  |  |
| Theoretical framework |  |  |
| 9. | Methodological orientation and Theory | Mixed methods evaluation comprised of pre/post-test questionnaire followed by thematic analysis of focus group data. |
| Participant selection |  |  |
| 10. | Sampling | Convenience sampling of 670 year one nursing & midwifery students at Queen’s University Belfast undertaking BSc Professional Nursing or BSc Professional Midwifery Degree. |
| 11. | Method of approach | Participants were approached by a gatekeeper (Director of Education) that was not associated with the study. |
| 12. | Sample size | 566 participants in phase one and 37 participants in phase two (qualitative aspect). |
| 13. | Non-participation | Participants were reminded that participating in this research would not affect their course grade. This was noted in the information sheet and within the consent form. |
| Setting |  |  |
| 14. | Setting of data collection | Focus group data was collected via online meetings (MS Teams). Data collection took place during the student’s own time. |
| 15. | Presence of non-participants | There were no non-participants present during the focus group interviews. |
| 16. | Description of sample | Participants were year one undergraduate nursing and midwifery students from Queen’s University Belfast. |
| Data collection |  |  |
| 17. | Interview guide | The interview guide was designed by the authors. |
| 18. | Repeat interviews | No repeated interviews were carried out. |
| 19. | Audio/visual recording | All focus group data was audio-recorded. |
| 20. | Field notes | No field notes were collected during focus group interviews. |
| 21. | Duration | Focus group interviews lasted approximately 40 minutes. |
| 22. | Data saturation | Data saturation was achieved in this study with the participation of 37 nursing and midwifery students in focus groups, wherein recurring themes and perspectives relevant to the podcast's impact on SDGs were consistently observed, indicating comprehensive exploration of the topic. |
| 23. | Transcripts returned | All participants had the option of reviewing their focus group transcript as noted in the information sheet, consent form and at the conclusion of the focus group interview. No participant requested this. |
| **Domain 3: analysis and findings** |  |  |
| Data analysis |  |  |
| 24. | Number of data coders | Qualitative data analysis, using thematic analysis (Braun and Clarke), was carried out by GM, SC & TA) |
| 25. | Description of the coding tree | All authors actively participated in the design, data collection, analysis, and interpretation of the study, ensuring comprehensive collaboration and shared contributions to the research process. |
| 26. | Derivation of themes | The authors used thematic analysis to analyse themes. This was led by GM, SC & TA initially and involved all team members in the advanced stages. |
| 27. | Software | The authors used NVivo Version 12 to manage the data. |
| 28. | Participant checking | No member checking was carried out. |
| Reporting |  |  |
| 29. | Quotations presented | Direct quotations were presented from participants while confidentiality was maintained. |
| 30. | Data and findings consistent | The authors have ensured there was concordance between the data and findings that are presented. All authors were involved. |
| 31. | Clarity of major themes | The authors have reported all major themes as identified in this research. |
| 32. | Clarity of minor themes | The authors were not required to report on any minor themes or deviant cases within their presentation of results as these did not emerge. |
